# Mechanical Properties of Lightweight Foamed Concrete Modified with Magnetite (Fe_3_O_4_) Nanoparticles

**DOI:** 10.3390/ma15175911

**Published:** 2022-08-26

**Authors:** Md Azree Othuman Mydin, Mohd Nasrun Mohd Nawi, Othman Mohamed, Marti Widya Sari

**Affiliations:** 1 School of Housing, Building and Planning, Universiti Sains Malaysia, Gelugor 11800, Penang, Malaysia; 2 Disaster Management Institute (DMI), School of Technology Management and Logistics, Universiti Utara Malaysia, Sintok 06010, Kedah, Malaysia; 3 Department of Quantity Surveying, Centre for Building, Construction and Tropical Architecture (BuCTA), Faculty of Built Environment, University of Malaya, Kuala Lumpur 50603, Malaysia; 4 Faculty of Science and Technology, Universitas PGRI Yogyakarta, Jl. PGRI I No. 117, Sonosewu, Yogyakarta 55182, Indonesia

**Keywords:** lightweight foamed concrete, magnetite nanoparticles, compressive strength, flexural

## Abstract

The advancement in sustainable construction has stimulated wide-ranging investigation of construction materials and practices globally. With exceptional thermal properties, fire resistance performance, excellent strength, and outstanding durability, concrete is the utmost extensively utilized construction material around the world. Taking into consideration the quantity of concrete necessary for numerous constructions works, improving concrete sustainability would be an extremely attractive potential. Lightweight foamed concrete (LFC) is tremendously permeable, and its mechanical properties weaken with a growth in the volume of voids. Air-void segregation from solid cement phases by means of aging, drainage, and merging of voids can trigger and reduce the stability and consistency of the emitted pores, making the LFC less reliable for main utilization in load-bearing components and structural elements. In turn, to augment LFC mechanical properties, the LFC cementitious matrix can be adjusted by adding various nanoparticles. The influence of magnetite nanoparticles (MNP) in LFC was not examined in the past; hence, there is some vagueness considering the mechanism to which level the MNP can affect the LFC mechanical properties. Thus, the aim of this study is to investigate the influences of MNP on the compressive, splitting tensile, and flexural LFC of 1000 kg/m^3^ density. Six MNP weight fractions of 0.10%, 0.15%, 0.20%, 0.25%, 0.30%, and 0.35% were considered. The parameters accessed were compressive, splitting tensile and flexural strengths. The correlation between strength parameters was established as well. The results indicated that a 0.25% weight fraction of MNP gave the best performance in terms of compressive, flexural, and splitting tensile strengths. The presence of MNP in the LFC matrix enhances the viscosity and yield stress of the mixture as well as an augmented utilization of LFC cementitious binder content, which can sustain the integrity of the wet networks hence preventing further amalgamation and aging of the voids.

## 1. Introduction

Rapid development has allowed the construction industry to continue thriving, but the challenges associated with the management of construction turn gradually more severe [1,2]. With the rising environmental challenges, it is vital that sustainable materials are explored for a broader range of applications to offer practical options alongside conventional materials. Additionally, with the fast expansion of the construction industry, the production of cement has risen promptly [3]; natural resources, for instance, the river sand, have plunged into scarcity [4]; and the use of energy and emissions of carbon dioxide have skyrocketed [5,6]. Hence, viable green building materials are considered necessary in the construction industry as an option [7]. Among a variety of choices, the use of lightweight foamed concrete (LFC) as building materials is deemed to be a prime approach. The extensive employment of LFC for roof insulation screed, load-bearing and non-load-bearing walls, floor slabs, trench reinstatement, ground stabilization, void filling, bridge abutments, and even a decorative panel has boosted the demand for cement production [8]. Due to the growth in infrastructure developments, the urging for concrete will rise in the near future [9]. With the ongoing development of housing and infrastructure projects, specifically the developing countries, the rate of utilization of cement and concrete is destined to expand further [10]. It is anticipated that the world cement fabrication will grow to about 4.8 billion tons per annum by the year 2040, causing an equal expansion in concrete production [11]. 

LFC is a cellular concrete with entrapped air by means of a suitable surfactant (foaming agent) that has increasingly become extensive because of its unique attributes such as wide ranges of density, free from coarse aggregates, and outstanding thermal performance [12]. LFC is generally self-leveling and self-compacting, and it can be pumped. It is made through blending, molding, maintaining, and hardening processes. Compared with other types of concrete, LFC contains a considerably higher pore volume, up to 80%, which substantially influences its insulation properties. The LFC cellular structure is created by combining suitable foaming agents that can be either protein or synthetic based [13]. The manufacturing of LFC is nontoxic, and the product does not produce toxic gases when it is exposed to fire. By properly controlling the foaming agent amount, LFC with densities ranging from 550 kg/m^3^ to 1850 kg/m^3^ can be achieved [14]. Additionally, the reduction in structural dead load due to its reduced density can aid in reining in the construction activity. As a reduced content of aggregate is used in the production of LFC, the total quantity of binder materials is usually greater than those used in normal concrete [15]. LFC is appropriate for constructing partition panels as filling materials and has been employed widely in the construction industry these days [16]. With the rapid development of the industrialized building system, it has a massive potential to adopt LFC for manufacturing partition walls for buildings. One of the drawbacks of the deployment of LFC in building construction is the low strength due to its porous matrix [17]. When LFC is used to precast building components, it needs a longer time to achieve the anticipated compressive strength compared to normal concrete [18]. 

To improve the stability and firmness of the created air bubbles, former investigations by Hou et al. [19] and Huang et al. [20] have demonstrated that growth in LFC viscosity by the inclusion of nano-size raw materials may keep the reliability of the wet networks in the cementitious composite. Lots of study works were carried out to enhance the cementitious matrix in LFC by augmenting the inventions of cement paste, such as the inclusion of natural and synthetic fibers, resins, fuel ash, and other types of additives [21]. Augmented void shape, uniformly dispersed air voids and their sizes, enhanced permeability [22], and reduced void sizes are all good approaches to enhancing the properties of LFC [23].

Nanotechnology incorporates exploitation of the structure at the nanoscale to create an invention of multifunctional and customized cementitious composites with outstanding durability and mechanical properties. Prospective unique properties of these materials comprise high ductileness, ability to control cracks formation, self-healing, minimal electrical resistance, self-detection, and self-cleaning. Nanoparticles are those with at least one dimension of less than 100 nm [23]. These days, nanoparticles have been receiving consideration and have been employed in many areas to produce advanced building materials with innovative purposes due to their exclusive chemical, durability, and physical properties. Nanoparticles can perform as a very dynamic artificial pozzolanic material to augment the performance of cement-based materials. The inclusion of nanoparticles in concrete may significantly enhance their compressive, flexural, and tensile strength strengths. Nanoparticles can serve as diverse nuclei for the cement paste, further speeding up the hydration of cement hydration owing to their superior reactivity as nano-fortification, densifying the concrete microstructure [24].

The application of nanoparticles in concrete is one of the few evolving areas. Titanium dioxide (TiO_2_), iron oxide (Fe_3_O_4_), aluminum oxide (Al_2_O_3_), carbon nanotubes, zirconium dioxide (ZrO_2_), and carbon nanofibers are the most frequently used nanoparticles in research [25]. These particles have shown a remarkable impact on the hydration of concrete due to a nucleation process in the cement matrix. They behave as a source for the hydration of calcium silicate hydrate [26]. Higher the rate of hydration, earlier is the strength gained in concrete. Same materials perform inversely with the reduction in the size of particles. Research has demonstrated that the addition of chemically inert nanoparticles, such as titanium dioxide (TiO_2_) and magnetite (Fe_3_O_4_), speeds up the early hydration by providing supplementary nucleation sites, higher rate peaks, and greater total heat of hydration [27]. Nanoparticles govern the atomic level matters, which deal with an atom size that is less than 100 nm [28,29].

## 2. Materials

### 2.1. Ordinary Portland Cement (OPC)

For this research, OPC with the label of CEM I 52.5 R, with a surface area and specific gravity of 3325 cm^2^/g and 3.09 correspondingly, has been employed as a binder to formulate the LFC. Table 1 reveals the physico-chemical properties of OPC utilized in this research. This OPC follows BS12-1996 [30].

### 2.2. Fine Sand

**Figure 1:**
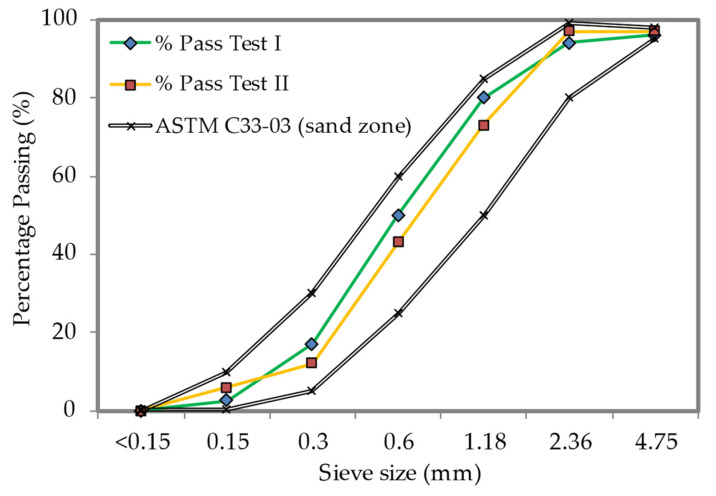
Fine sand particle size distribution.

Fine river sand with additional sieving to eliminate particles larger than 2.36 mm was utilized in the LFC mix to enhance the LFC flowability and stability as described in BS12620-2013 [31], a 60–90% passage through a sieve of 600-micron. The fine sand was provided by a local distributor. Figure 1 indicates the sieve analysis chart of fine sand employed in this research.

### 2.3. Surfactant

A protein foaming surfactant called Noraite PA1 was employed in this laboratory-based appraisal. The properties of this surfactant are presented in Table 2. The protein foaming surfactant was diluted with water at a proportion of 1:34 by volume. Next, a foam maker was utilized along with the air compressor to generate stable foams. The air pressure was maintained between 400 and 450 kPa, which was employed to create stable foam density between 70 and 80 g/L.

### 2.4. Water

The laboratory works utilized clean tap water, which was free from organic elements or detritus. The water-cement proportion was kept constant at 0.45 because it gave reasonable workability. The tap water was utilized to make the mortar slurry, LFC mixing, and curing purposes.

### 2.5. Magnetite Nanoparticles

**Figure 2:**
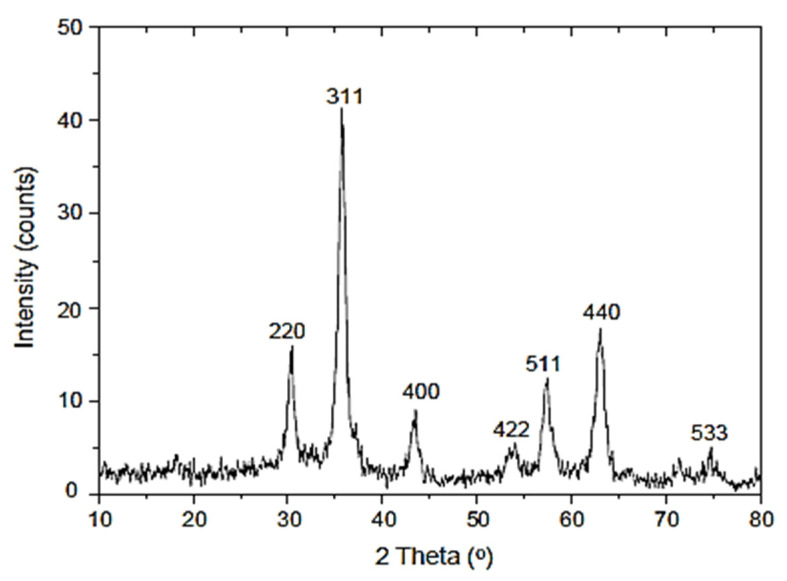
MNP X-ray diffraction patterns.

**Figure 3:**
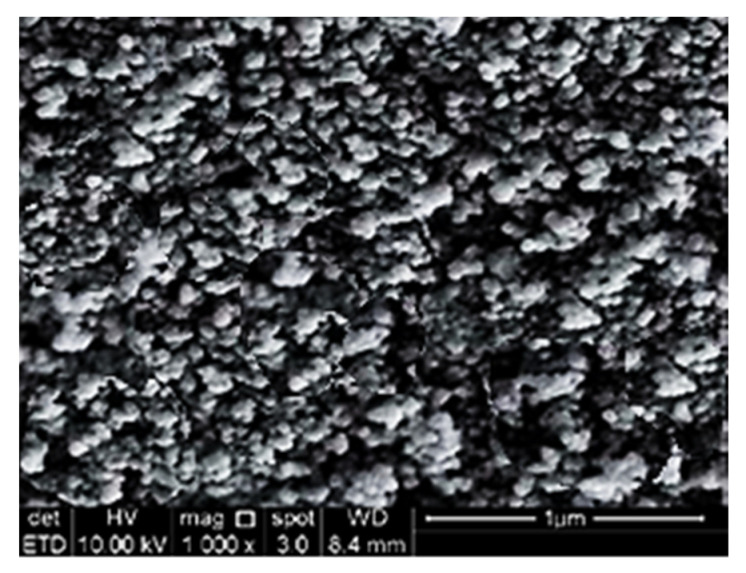
Morphology of pure MNP.

Magnetite (Fe_3_O_4_) nanoparticles with grain sizes of 40–60 nm were employed, which had a purity of more than 99% and were supplied by DRN Technologies Sdn Bhd. There were 6 different weight fractions of MNP were employed which were 0.10%, 0.15%, 0.20%, 0.25%, 0.30% and 0.35%. Figure 2 illustrates the X-ray diffraction patterns for magnetite nanoparticles (MNP) used in this study. Six maximum peaks at 2θ of 31°, 37°, 44°, 54°, 58°, and 64° with narrow and well-defined peaks can be attributed to the spacing of crystal planes of the typical cubic MNP. From the diffraction patterns, a sequence of characteristic peaks (215), (306), (396), (418), (505), and (437) were detected, which is similar to the typical MNP diffraction spectrum (JCPDS file no: 19-0629) which acknowledged the crystallographic system of cubic structure [32]. A vast disparity can be obviously noticed in that the XRD patterns of maghemite comprise many peaks, unlike magnetite, which only entails a few peaks. Figure 3 demonstrates the morphology of pure MNP utilized in this study.

**Figure 4:**
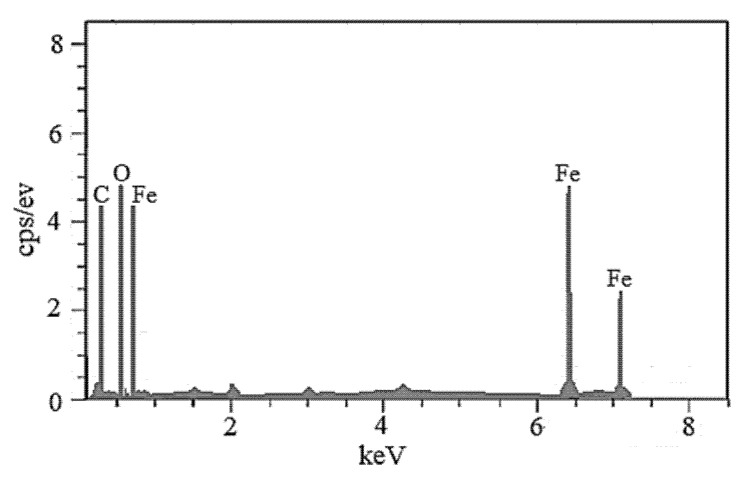
Energy dispersive spectrometry (EDS) of MNP.

Figure 4 demonstrates the elemental composition of MNP, which was determined via Energy Dispersive Spectrometry (EDS). Table 3 summarizes the elemental compositions of MNP. The existence of an extreme peak of Fe confirms the formation of magnetite nanoparticles.

Next, the FTIR spectroscopy procedure was executed to determine the functionalization of the MNP. Table 4 demonstrates the FTIR peaks of MNP. It can be seen that a thick peak around 498 cm^−1^ reflects the ferrous oxide (Fe-O) bond absorption. This validates the existence of the magnetic core, and therefore, it is more prominent in the bare MNP. The band around 2897 cm^−1^ is designated for stretching and vibration of –CH_2_ groups, signifying the existence of oleic acid. Hence, the FTIR results verify the functionalization of the MNP. The band at about 1591 cm^−1^ signifies the stretching and vibration of the H–C=O backbone. The absorption peak around 3389 cm^−1^ is discovered, which may be originated from hydroxyls (OH) appearing in water and signaling the existence of the polysaccharide shell.

## 3. Mix Design and Specimen Preparation

There were seven LFC mixtures of 1000 kg/m^3^ density were manufactured. The MNP weight fractions utilized in this investigation was 0.10%, 0.15%, 0.20%, 0.25%, 0.30%, and 0.35%. Sand-cement proportion of 1:1.5 was employed, while a 0.45 water-cement proportion remained constant at 0.45 for the entire mixture. The mix design of LFC is displayed in Table 5.

## 4. Test Methods

### 4.1. Compression Test

**Figure 5:**
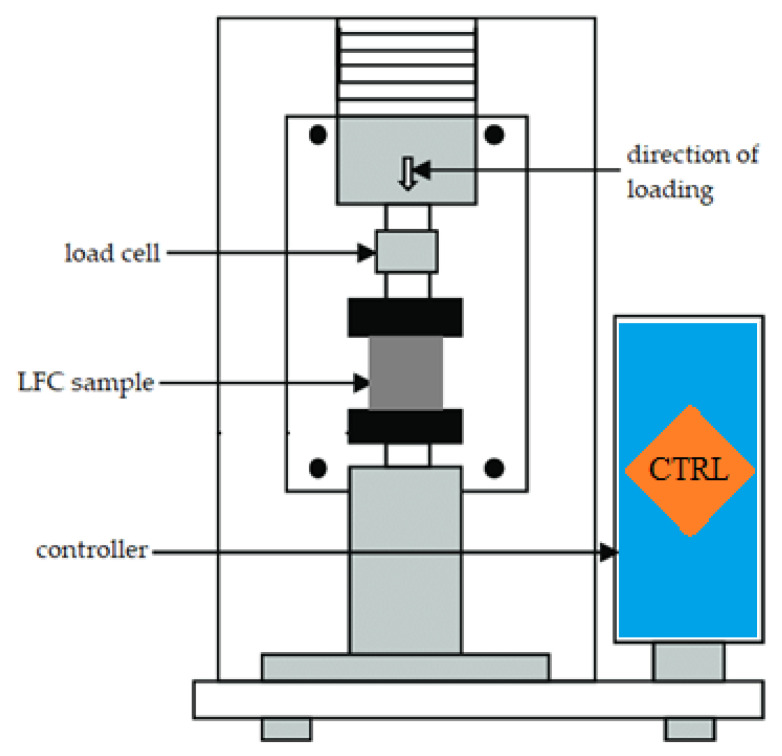
Schematic diagram of the compression test setup.

The compression test was performed in conformity with BS-EN12390-3 [33] on a 100 × 100 × 100 mm cube. Figure 5 shows the schematic diagram of the compression test setup. In each case, the results quoted are the average of three specimens. An axial compressive load with a constant pace of 0.025 mm/s was employed on the LFC cubic samples. The LFC compressive strength is established using the subsequent formula:

Compressive strength = P/A(1)

where

P = upper limit load incurred by the LFC sample (N)
A = LFC sample cross-sectional area (mm^2^).

### 4.2. Flexural Test

**Figure 6:**
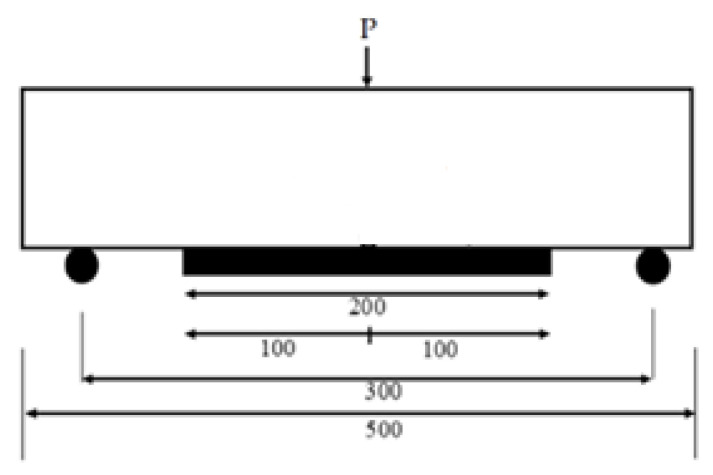
Schematic diagram of the LFC sample geometry and setup for flexural test.

The flexural test was executed conforming with BS EN 12390-5 [34] on a prism specimen with a size of 100 × 100 × 500 mm. Flexural strength tests for LFCs were performed in triplicate, but only the average readings were stated. The test was accomplished with center-point loading (Figure 6). A focused load, P in terms of a stroke at the constant rate of 0.085 mm/s, was imposed at the mid-span. During this period, the force that instigated fracture was concurrently observed. The flexural strength of the LFC prism specimen is determined using the following equation.

Flexural strength = 3PL/2bd^2^ (2)

where

P = maximum load sustained by the LFC specimen (N)
L = span length (mm)
b = width of tested prism (mm)
d = depth of tested prism (mm).

### 4.3. Splitting Tensile Test

**Figure 7:**
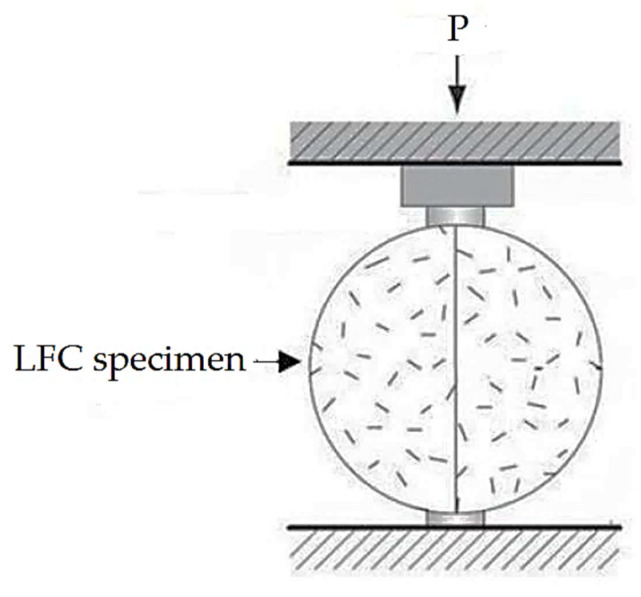
Schematic diagram of splitting tensile strength test.

The splitting tensile strength test in this research was accomplished in line with BS EN 12390-6 [35]. A cylinder LFC sample of 100 mm in diameter and 200 mm in height was employed for this test. Figure 7 indicates the schematic diagram of the splitting tensile strength test. The splitting tensile strength is established using the following equation:

Splitting tensile strength = 2P/ΠDL(3)

where

P = maximum load sustained by the LFC sample (N)
D = diameter of test cylinder(mm)
L = length of the test cylinder (mm).

## 5. Results and Discussion

### 5.1. Compressive Strength

**Figure 8:**
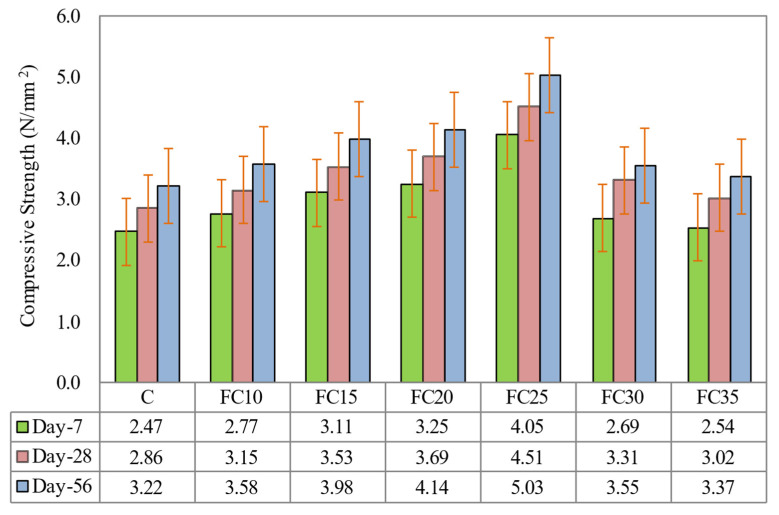
Influence of different weight fractions on compressive strength of LFC.

Figure 8 illustrates the compressive strengths of all the LFC specimens at the ages of 7, 28, and 56 days. Based on Figure 8, it is obvious that the presence of MNP in LFC had increased the compressive strength for entire mixes and testing ages compared to specimen C (control sample). The optimal compressive strength was obtained with the presence of 0.25% MNP in the LFC mix (specimen FC25), which gave an improvement of 64.0%, 57.7%, and 54.6% at curing ages 7, 28, and 56 days correspondingly in comparison to the control sample (Specimen C). There were two reasons that may be responsible for the encouraging impact of MNP inclusion on the compressive strength of the LFC. Primarily, it is recognized that the MNP fine unit size in LFC cementitious matrix can substantially alter the kinetics of hydration of the matrix [36]. Thus, MNP fine particle size will play an important role in accelerating the cement hydration process due to their high level of activity. They provide significant reactive surfaces, which may act as a nucleation site, thus stimulating the nucleation responses of hydration phases nuclei on their surface. As the accelerating effects of these particles are produced by surface reactions, the surface area or the size of the particles, respectively, are the main factors for the particle’s effectiveness in controlling the cement hydration kinetics. The other possibility to quicken cement hydration is the addition of MNP, which themselves are the nuclei of hydration phases such as C-S-H seeds. In this case, the composition of the nuclei has to be the same as the demanded hydration phase. By the addition of these particles, the time-consuming nucleation reactions, which are necessary for further crystal growth, can be prevented, and the inactive period of cement hydration is reduced. As the C-S-H growth during the acceleration period is autocatalytic, the formation of C-S-H in this period should be boosted considerably due to the presence of the added C-S-H particles.

**Figure 9:**
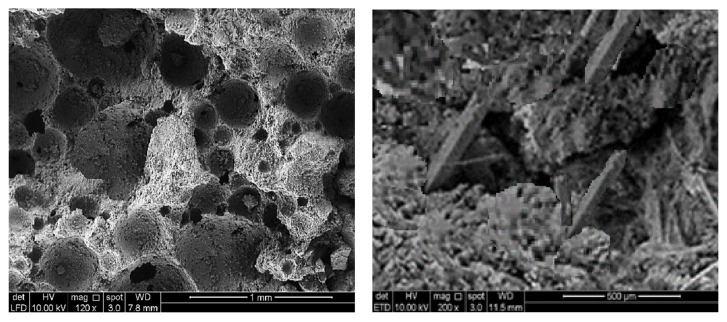
Morphology of plain LFC (**left**) and MNP-LFC composites (**right**).

Figure 9 shows the morphology of LFC and MNP–LFC composites. The inclusion of MNP had an effective role in creating additional interlocked CSHs needles, which strengthened the microstructure of LFC. This was explained by Amin et al. [37], where a small volume fraction of nanoparticles of 0.3% was employed. While the hydration process occurs, the hydrate products dispersed and encircled the MNP, behaving as the grain. Secondly, owing to their fine size, the MNP fills the voids, leading to the further compacting of the LFC microstructure [38]. If the volume fraction of the nanoparticles is optimal, the crystallization will be regulated, and the expansion of calcium hydroxide crystals will be inhibited by the MNP. Khoshakhlagh et al. [39] performed a study on high-strength concrete to observe the consequences of Fe_2_O_3_ on the strength and permeability properties. The research implemented numerous tests such as compression, flexural, and water permeability tests. It was shown that the compressive strength and water permeability expanded with the presence of 4% Fe_2_O_3_ nanoparticles.

Nevertheless, when the volume fraction of magnetite nanoparticles surpasses the necessary limit, calcium hydroxide crystals cannot rise up sufficiently due to the restricted space in the cementitious matrix. The reduced proportion of the crystals to the fortifying gel leads to an expansion of creep and shrinkage in the LFC cementitious matrix. Accordingly, the pore form of the LFC matrix is loose [40]. These two main reasons led to the enhancement of the LFC microstructure by decreasing the number of voids, expanding the connection between the fine filler (sand) and the cement matrix, and boosting the density of the LFC. The decrease in compressive strength with a further increase in the MNP weight fractions from 0.30% to 0.35% was ascribed to the accumulation of MNP at higher weight fractions which had an adverse effect on the hydration process and the bonding strength of LFC. Overall, the inclusion of MNP increases the pore structure of LFC [41].

### 5.2. Flexural Strength

**Figure 10:**
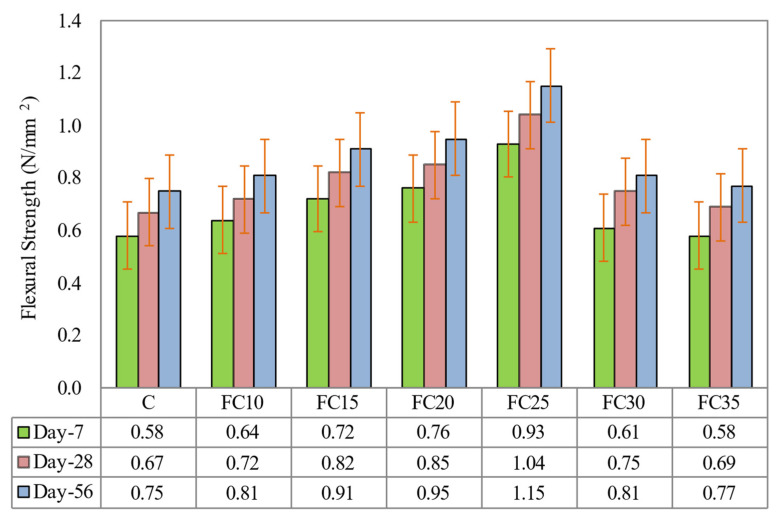
Influence of different weight fractions on flexural strength of LFC.

Figure 10 validates the flexural strengths of all the LFC specimens at the ages of 7, 28, and 56 days. According to Figure 10, it can be seen that the existence of MNP in LFC had enhanced the flexural strength for entire mixes and testing ages compared to specimen C (control sample). The optimal flexural strength was accomplished with the presence of 0.25% MNP in the LFC mix (specimen FC25), which gave an enhancement of 60.3%, 55.2%, and 53.3% at curing ages 7, 28, and 56 days, respectively, in comparison to the control sample. The greater flexural strength attained with the inclusion of MNP was due to the prompt utilization of crystalline calcium hydroxide, which immediately arises throughout the hydration of cement, specifically at an early age because of the high reactivity of MNP.

Subsequently, the hydration of cement is augmented, and greater volumes of reaction products are created [42]. Likewise, MNP recuperates the LFC packing density, leading to a decreased volume of greater pores. Additionally, the enhancement in LFC flexural strength was due to further hydration of cement as MNP has high reactivity by behaving as a core for cement segments. Moreover, it also acts as a nano-fortification element and accordingly densifies the LFC microstructure and interferential transition zone, reducing the porosity and filling the voids to boost the LFC flexural strength.

Nazari et al. [43] studied the impact of the integration of ferric oxide (Fe_2_O_3_) nanoparticles up to a maximum replacement of 2% on flexural and tensile strengths of concrete, and they found that nano-Fe_2_O_3_ particle’s presence in concrete had significantly led to higher flexural strength and reduced setting time compared to the control specimen. Additionally, the flexural strength improved with a rise in the volume fraction of Fe_2_O_3_ nanoparticles. Fe_2_O_3_ nanoparticles reduce the total porosity of the concrete, alter the pore structure of the cement matrix, and substantially diminish the permeability of LFC [44].

As discussed in the previous section, the improvement in the flexural strength of LFC with an increase in the MNP weight fraction up to 0.25% was because of the reinforcement effect of MNP together with their uniform spreading in the cement matrix, which results in a more compact microstructure compared to that of control specimen. However, at an MNP weight fraction higher than 0.25%, the magnetite nanoparticles were agglomerated with inadequate distribution, which adversely impacted the bonding strength of the composite.

### 5.3. Splitting Tensile Strength

**Figure 11:**
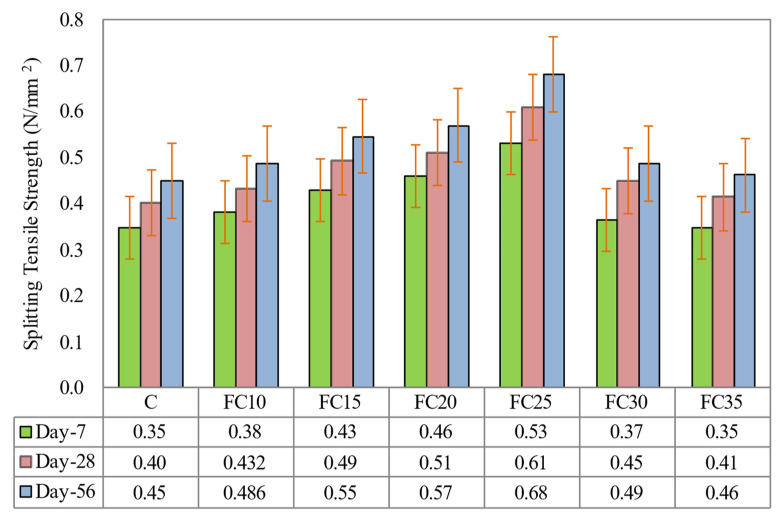
Influence of different weight fractions on splitting tensile strength of LFC.

Figure 11 demonstrates the splitting tensile strengths of all the LFC at the ages of 7, 28, and 56 days. It was discovered that the splitting tensile strength of LFC improved with augmented weight fraction of MNP, as seen in Figure 10, signifying that the existence of nanoparticles in a cementitious matrix of LFC plays a vital role in accelerating the process of hydration in comparison to control LFC samples. These atoms are extremely active and volatile, causing a speedier hydration process. The optimal splitting tensile strength was accomplished with the presence of 0.25% MNP in the LFC mix (specimen FC25), which gave an enhancement of 51.4%, 52.5%, and 51.1% at curing ages 7, 28, and 56 days, respectively, in comparison to the control sample.

The remarkably small surface area of MNP permeates the cavities in the hydrated cementitious matrix, which amasses to a greater density and consequently results in greater splitting tensile strength of LFC. The mechanism by which the MNP addition in LFC improves the pore structure of cement matrix was evenly scattered in LFC, and each particle is enclosed in a cube pattern; thus, the gap between MNP can be revealed [45]. Once the hydration commences, hydrate products are disseminated and encase MNP as kernels.

If the MNP content and the distance between them are appropriate, the crystallization will be regulated by impeding the expansion of calcium hydroxide crystals by MNP. Subsequently, the pore structure of the LFC is obviously enhanced, such as the LFC containing an MNP of 0.25% by weight fraction. The decrease in splitting tensile strength by adding more than 0.25% weight fraction of MNP was because the quantity of MNP appearing in LFC is greater than the quantity necessary to merge with the cementitious matrix [46,47].

### 5.4. Performance Index (PI)

**Figure 12:**
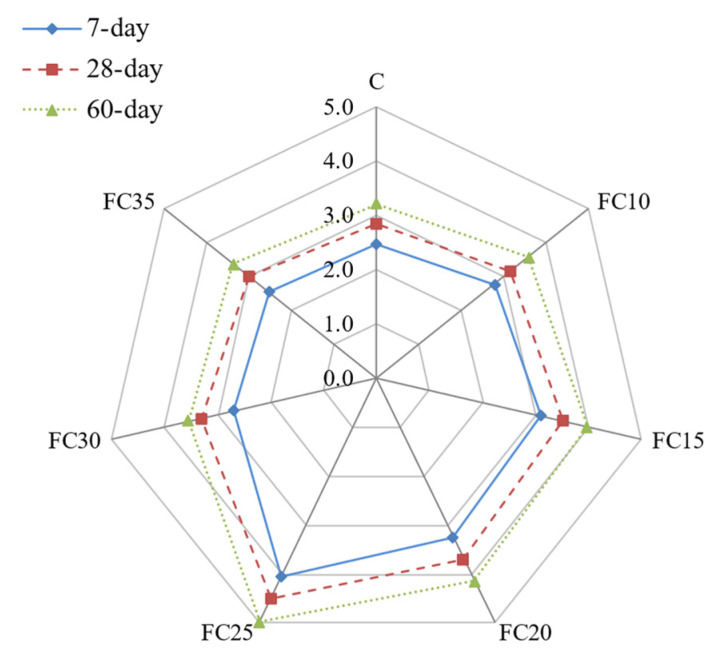
Influence of different weight fractions on performance index of LFC.

The LFC compressive strength and density have a correlated relationship. Theoretically, the greater the density of LFC, the greater will be the compressive strength. The density of LFC for this research was controlled at 1000 kg/m^3^. As the density for each specimen was slightly varied with the addition of MNP, the performance index of LFC was evaluated to boost the accuracy of the results accomplished through the laboratory investigation. Figure 12 shows the PI calculated and charted. A similar trend was acquired by the PI, in which the PI is precisely comparative to the specimen’s curing age. The highest 56-day PI was achieved by LFC blended with a 0.25% weight fraction of MNP, which is 4.98 N/mm^2^ per 1000 kg/m^3^. It is acknowledged that the fine particle of MNP can substantially modify the hydration process of mortar slurry.

Due to their stiffer electrostatic forces and outstanding specific surface area, a quicker setting time and hardening of the LFC composites can be achieved. Hence, MNP fine particle size will play a major role in accelerating cement hydration owing to their high level of activity. When the hydration process began to appear, the hydrate products scattered and encompassed the MNP acting as the grain [48]. Furthermore, due to MNP’s fine size, the MNP fills the cavities, leading to the additional packing of the LFC microstructure [49]. When the volume fraction of the magnetite nanoparticles added into LFC mixes is ideal, the crystallization will be synchronized [50], and the growth of calcium hydroxide crystals will be impeded by the MNP.

### 5.5. Relationship between Compressive and Flexural Strengths

**Figure 13:**
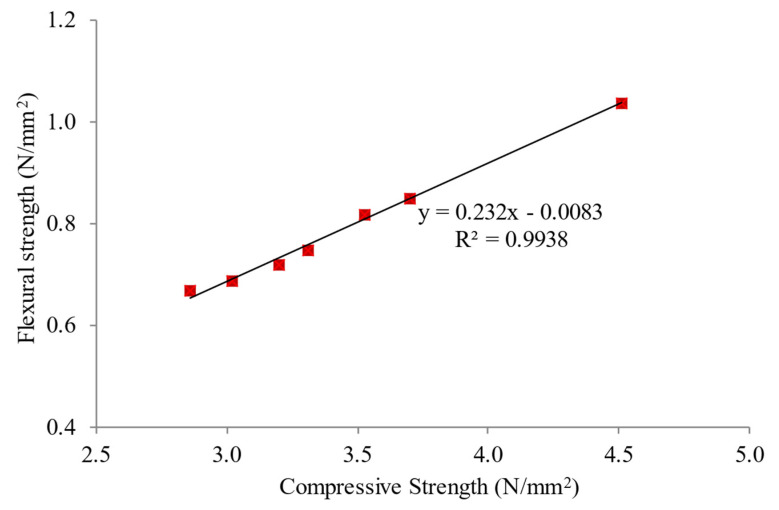
Relationship between compressive and flexural strengths of LFC.

Figure 13 demonstrates the possible association between the compressive strength and the flexural strengths of LFC with the inclusion of MNP. Compressive strength was plotted against flexural strength. According to Figure 13, the distribution of data confirms that a decent connection between compressive strength and flexural does exist. A strong linear correlation is evident with a high regression value (0.9938). For the control specimen (plain LFC), the flexural strength was about 20% of the compressive strength of the LFC. The flexural strength compounded up to an average of 29% of the compressive strength with the ideal weight fraction of 0.25% MNP. The reason for this increase in flexural strength is associated with the pozzolanic activity of the LFC cementitious matrix as well as their filling effects with the presence of MNP [51]. It enhanced the microstructure of cement paste through their reaction with CH and the production of secondary CSH as MNP has superior reactivity by acting as a nucleus for cement portions [52]. MNP recaptures the LFC particle filling density [53], consequently diminishing the number of pores in the cementitious composite [54].

### 5.6. Relationship between Compressive and Splitting Tensile Strengths

**Figure 14:**
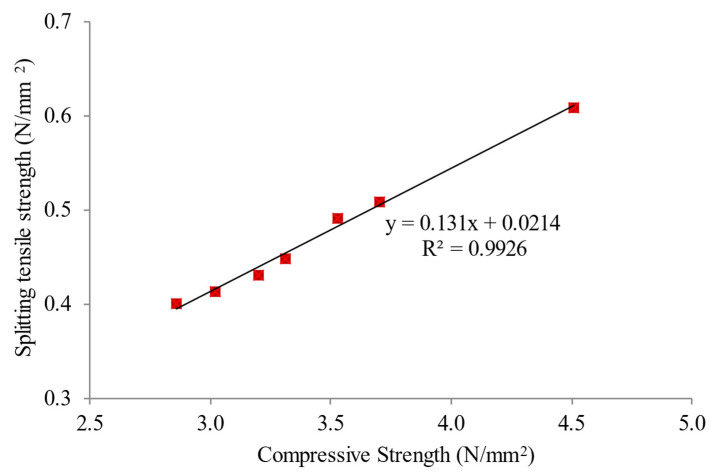
Relationship between compressive and splitting tensile strengths of LFC.

Figure 14 visualizes the association between compressive and splitting tensile strengths of LFC. It evidently revealed that a direct increasing pattern can be recognized in splitting tensile strength versus compressive strength, with an R-squared value in the order of 0.9926. The connection explains that splitting tensile strength increases with increasing compressive strength. The mechanism by which the MNP inclusion in LFC enhances the pore structure of the cementitious matrix was uniformly dispersed, and each particle was confined in a cube pattern; thus, the disparity between MNP can be discovered. As soon as the hydration initiates, hydrate products are distributed and enclosed in the MNP as kernels. Additionally, the MNP presence in the LFC matrix as kernels can help to stimulate cement hydration due to their high responsiveness [55].

## 6. Conclusions

In this investigation, LFC was incorporated with 0.10–0.35% MNPs by weight fraction. Mechanical properties such as splitting tensile strength, compressive strength, and flexural strength were assessed. Based on the studies, the following conclusions are drawn.

1. MNP has an excellent potential to be incorporated with LFC for mechanical properties enhancement due to its distinctive properties, such as high ductileness and the ability to control crack growth. The inclusion of MNP in LFC enhanced the compressive, splitting tensile and flexural strengths significantly. The optimal results for these three mechanical properties were attained with specimen FC25.
2. The optimal compressive strength was obtained with specimen FC25, which gave an improvement of 64.0%, 57.7%, and 54.6% at curing ages 7, 28, and 56 days, respectively, compared to the control specimen. The inclusion of MNP had an effective role in creating additional interlocked CSHs needles, which strengthened the microstructure of LFC.
3. The highest flexural strength was accomplished with specimen FC25, which gave an enhancement of 60.3%, 55.2%, and 53.3% at curing ages 7, 28, and 56 days, respectively, in comparison to the control sample. The greater flexural strength attained with the inclusion of MNP was due to the prompt utilization of crystalline calcium hydroxide, which immediately arises throughout the hydration of cement, specifically at an early age because of the high reactivity of MNP.
4. The ideal flexural strength was achieved with specimen FC25 which gave an improvement of 51.4%, 52.5%, and 51.1% at curing ages 7, 28, and 56 days, respectively, in comparison to the control specimen. The remarkably small surface area of MNP permeates the cavities in the hydrated cementitious matrix, which amasses to a greater density and consequently results in greater splitting tensile strength.
5. The reduction of compressive, flexural, and splitting tensile strengths with a further rise in the MNP weight fractions from 0.30% to 0.35% was attributed to the accretion of MNP at higher weight fractions, which had an inauspicious result on the hydration process and the LFC bonding strength.
6. Owing to MNP’s fine size, the MNP fills the cavities, leading to the additional packing of the LFC microstructure. When the volume fraction of the MNP added into LFC mixes is ideal, the crystallization will be synchronized, and the growth of calcium hydroxide crystals will be impeded by the MNP.

## Figures and Tables

**Table 1:** Physico-chemical properties of OPC.

Elements	Percentage (%)
CaO	65.15
SiO_2_	15.81
Al_2_O_3_	4.03
MgO	1.23
SO_3_	3.85
Fe_2_O_3_	6.87
Na_2_O	0.49
Insoluble residue	1.22
LOI	1.35
Setting time (Initial/Final)	170/215
Specific surface area (cm^2^/g)	3325
Specific gravity	3.09
28 days Compressive Strength (N/mm^2^)	51.8

**Table 2:** Properties of synthetic-based surfactant.

Elements	Properties
Density (g/cm^3^)	1.18
Appearance	Light brown
pH	6.2
Specific gravity	1.09
Dilution ratio	1:34
Molar mass	265 g/mol

**Table 3:** Elemental compositions of MNP.

Elements	MNP Weight (%)
Iron	65.85
Carbon	28.22
Oxygen	5.93

**Table 4:** FTIR peaks of MNP.

Types of Bond	Wavenumber (cm^−1^)	Remarks
OH stretching	3389	The presence of hydroxyls (OH) in water and polysaccharides shell
–CH_2_ stretching and vibration	2897	The presence of oleic acid
H–C=O stretching and vibration	1591	Extending of H–C=O backbone
Fe-O stretching	498	Ferrous oxide bond absorption

**Table 5:** LFC mix design.

Sample Code	Dry Density (kg/m^3^)	MNP Weight Fraction (%)	MNP (kg)	Cement (kg)	Fine Sand (kg)	Water (kg)
C	1000	0.00	0.000	37.47	56.20	16.86
FC10	1000	0.10	0.114	37.47	56.20	16.86
FC15	1000	0.15	0.170	37.47	56.20	16.86
FC20	1000	0.20	0.227	37.47	56.20	16.86
FC25	1000	0.25	0.284	37.47	56.20	16.86
FC30	1000	0.30	0.341	37.47	56.20	16.86
FC35	1000	0.35	0.398	37.47	56.20	16.86

## Data Availability

Not applicable.
